# Females occasionally create duets with males but they never sing solo-year-round singing behaviour in an Afrotropical songbird

**DOI:** 10.1038/s41598-023-38552-5

**Published:** 2023-07-14

**Authors:** Michał Budka, John Emenike Uyeme, Tomasz Stanisław Osiejuk

**Affiliations:** Department of Behavioural Ecology, Faculty of Biology, Adam Mickiewicz University in Poznań, Uniwersytetu Poznańskiego 6, 61614 Poznań, Poland

**Keywords:** Behavioural ecology, Animal behaviour, Tropical ecology

## Abstract

Our knowledge of birdsong mainly comes from studies focused on male songs produced in a short breeding period, even though we know that sedentary species sing year-round, female song is quite widespread and many species sing collectively creating duets and choruses. In this study we focused on daily and seasonal changes in singing activity of an endemic, sedentary, duetting, Afrotropical songbird—the Bangwa forest warbler. We collected soundscape recordings in six recording locations and used singing activity index to examine how vocal activity of males and females varies daily and seasonally and how it correlates with the rainfall. We found that Bangwa forest warblers sing year-round, yet they do it more in wet than in dry season. The rapid increase of singing activity occurs after first rain, at the beginning of the rainy season. Males sing significantly more than females. Females never sing solo, however, in 13% of songs they create duets by joining male solos. The pattern of daily singing activity is sex-specific and seasonally variable, with two peaks (dawn and dusk) observed in males and only one in females (dawn). In Bangwa forest warbler male singing behaviour is similar to that of many songbirds, suggesting that territory defence and female attraction as main functions of singing. Females, which create duets and never sing solo may use songs in mate guarding, signalling commitment, resource defence or intersex territory defence. Duets observed year-round may suggest cooperative resource defence. Results of the study show that examining year-round singing behaviour is crucial to fully understand the evolution and functions of male and female songs.

## Introduction

Initially birdsong has been defined as a long, complex vocalization produced by males in the breeding season to defend territory and attract females^1^. Such state of knowledge was the result of a large disproportion between bioacoustics studies conducted mostly in temperate and rarely in tropical regions. Indeed, in temperate regions predominantly males sing, and the peak of their vocal activity is observed at the beginning of the short breeding season—when males establish territories and attract females^1,2^. In many species singing activity decreases at a stage of incubation and parental care for chicks, to increase once more before the next brood, when females are fertile again^3^. In daily context, most bird species sing most intensively around sunrise (dawn chorus) and sunset (dusk chorus), however songs, with various singing intensity, are produced over the whole day, as well as at night in some species^4–7^.

In contrast to temperate regions, many tropical bird species are sedentary, defend their territories year-round but breed in different times of the year, depending on food availability and weather conditions^8,9^. Therefore, presence of singing throughout the whole year should be expected, while seasonal peaks of vocal activity should be a good indicator of reproductive activity^10^. Unfortunately, studies analysing year-round changes in vocal activity are very rare, even though they may help fully understand the biology, ecology and behaviour of birds^9,11–13^, as well as improve monitoring methods^14^. Using autonomous sound recorders, which enable sampling the soundscape over the whole year, may help to fill these knowledge gaps, especially in regions and habitats which are difficult to access in some parts of the year.

An overview of birdsong at a global scale reveals that female song is quite widespread, especially in the tropics, where it has been observed in over 70% of songbird species^15^. Female song appears to be the ancestral state^15^, and the rare song performance by females in temperate species may be the effect of rapid loss of female singing ability^16^. However, understanding evolution and functions of female song needs further studies, examining this phenomenon in biologically and ecologically diverse species, as well as both during and outside of the breeding season^17^. Concentrating only on breeding season may lead to misleading conclusions or even overlooking of female song. For example, in European robin *Erithacus rubecula* females sing and defend only the wintering territories, whereas they remain silent in the breeding season^18^. In Alpine accentor *Prunella collaris*, females sing only during their fertile period to attract mates, signalling quality and receptivity for mating^19^.

In many bird species the male and female sing collectively in a coordinated way, creating duets^20^. Duets have been observed in over 16% of bird species^21^ and show great variation between duetting species^22^. Males and females may sing cooperative songs in antiphonal or simultaneous manner, with various level of coordination. The phrases produced by males and females may be simple or complex, sex-specific, or indistinguishable between the sexes^20^. Depending on species, a duet may be initiated by the male, female or both sexes in various proportions^23^, and the proportion of duets and solos may vary considerably both between as well as within a species^22^. Evolution of duets is strongly associated with the coordinated year-round defence of ecological resources by both males and females^21^. The demand for and availability of resources is seasonally variable^24^. Therefore, we may expect a sex-specific and seasonally variable motivation for defence of resources, which should also be reflected in yearly changes in singing activity of males and females^11^. However, most of the studies on duet functions have been focused on the breeding or pre-breeding season, while only few have examined collective singing in year-round context (e.g.:^11,25–27^), suggesting seasonally-variable and species specific duetting behaviour^25–27^. Therefore, we do not know a lot of how often and in what context birds use duets outside the breeding season, even though this knowledge is essential to forming hypotheses about evolution and function of collective singing.

In the study we focused on year-round singing behaviour in Bangwa forest warbler *Bradypterus bangwaensis*—a sedentary, tropical, endemic, duetting songbird found in the Bamenda Highlands, a biodiversity hotspot located along the Cameroon and Nigeria border^28^. The Bangwa forest warbler is a small, territorial bird without sexual dimorphism, found in various montane-forest and bush habitats characterised by dense undergrowth. The distribution range of Bangwa forest warbler covers approximately 6900 km^2^ in the highlands (from 1600 to 2950 m asl) of west Cameroon and south-east Nigeria^29^. The worldwide population size is unknown, but the species is locally common, and is thus classified as Least Concern on the IUCN Red List of Threatened Species (BirdLife International 2022). The biology and ecology of Bangwa forest warblers have been poorly studied. It breeds mainly in October and November, with a possible second brood in March and April, however no detailed studies on the breeding phenology have been conducted yet. Males produce a characteristic song, in which they repeat an identical note, increasing in amplitude^29,30^. Females may create duets by singing whistles which decrease in frequency. Duetting has been also found in another 5 of the 12 species from genus *Bradypterus* (Family Locustelidae)^31^. However, to the best of our knowledge, there have been no studies focused on singing behaviour of any *Bradypterus* species, including the seasonal changes in vocal activity and duet function.

In this study we analysed year-round soundscape recordings collected with autonomous sound recorders in six recording locations to describe daily and seasonal changes in singing activity of the Bangwa forest warbler. We examined (1) when the daily and seasonal peaks of vocal activity occur; (2) how common are female songs and duets, and by which sex they are initiated; (3) whether the proportions between solos and duets change daily and seasonally; and (4) whether general singing activity correlates with precipitation. Looking at daily and seasonally changing proportions between solos and duets we propose potential hypotheses explaining the functions of duets and female song. Additionally, we demonstrate the importance of analysing year-round singing activity to fully understanding animal biology, ecology and behaviour, and show that autonomous sound recorders can be a powerful tool in such studies.

## Results

**Figure 1:**
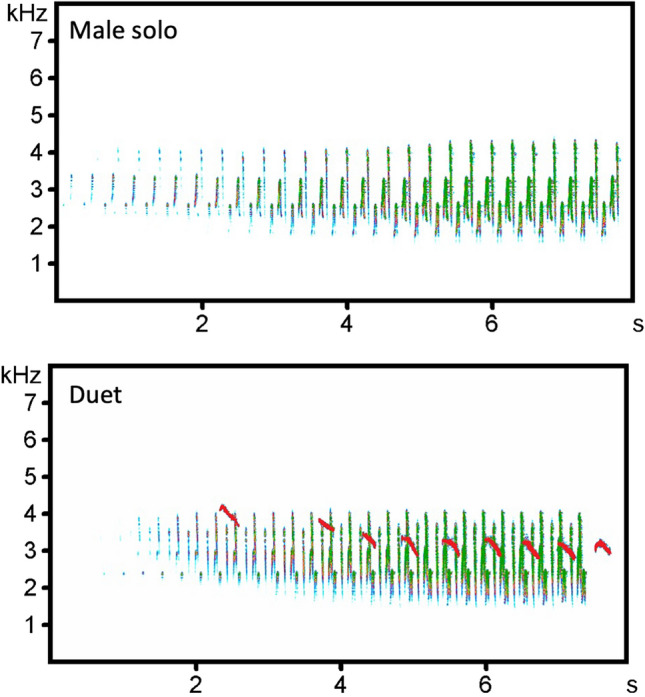
Spectrograms showing male solo song and duet. Males repeat identical notes (green colour), increasing in amplitude (darker green means higher amplitude). Females (red colour) create the duet by singing whistles which decrease in frequency. Spectrograms created in Avisoft-SAS Lab Pro 5.2.09 software. Recording was downsampled to 16 kHz. The colour of female syllables was manually modified to improve visibility. Original recordings are available as Supplementary Audio 1 and Supplementary Audio 2.

We collected soundscape recordings at six recording locations every seventh day over the whole year and analysed 1 min sound samples (1 min every 15 min, from one hour before sunrise to one hour after sunset) to describe the Bangwa forest warble singing behaviour. We found 11,856 songs of Bangwa forest warbler in 3058 1 min sound samples, from which 1546 were duets (13%) and 10,310 were male solos (87%) (Fig. 1; Supplementary Audio 1, Supplementary Audio 2). All duets were created by females that joined males singing solo. We have not recorded choruses (i.e., when more than two individuals sing together in coordinated way) or female solo songs. The Bangwa forest warbler songs were recorded at each recording location (from 1118 to 2953 songs per location) and during each recording day (from 24 to 330 songs per recording day). Depending on the recording location, duets represented from 7 to 17% of recorded songs (see Supplementary materials S1 Table for more details).

### Seasonal changes in vocal activity

**Figure 2:**
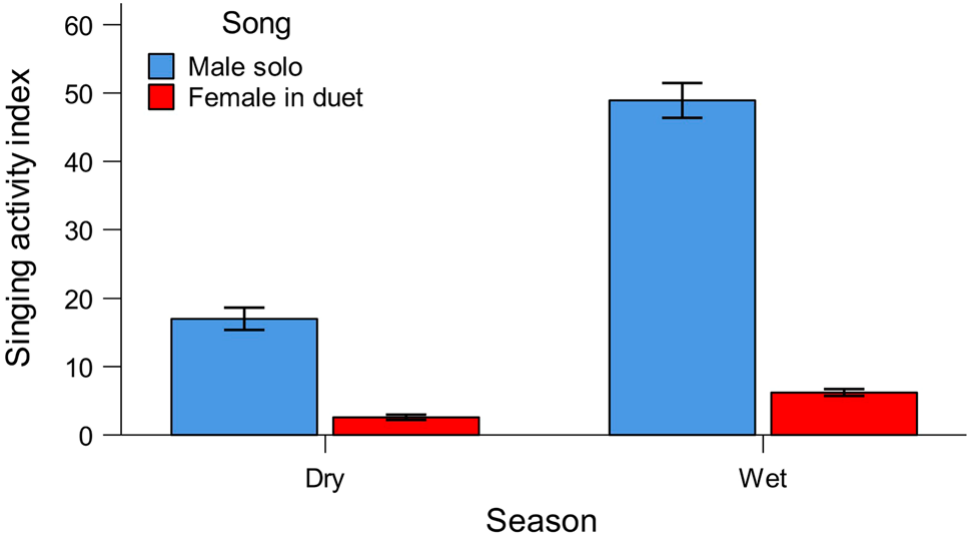
Daily singing activity index (mean +/− SE number of songs detected in 56–58 1 min sound samples per recording location per day) of males and females in dry (Nov–Feb) and a wet (Mar–Oct) season. Females never sing solo, therefore female songs are identical to duet. Significant differences are observed between males and females (*p* < 0.001), dry and wet season (*p* < 0.001) and interaction between sex and season (*p* < 0.01). See Table 1 for more details.

Bangwa forest warbler sang significantly more in wet than in dry season (daily singing activity index: 27.1 ± 32.82 versus 9.9 ± 13.69 songs per day per recording location, respectively). Males produced significantly more songs than females (daily singing activity index: 38.0 ± 33.42 versus 5.0 ± 6.23 songs per day per recording location, respectively; Fig. 2). Interaction between sex and season was significant, which means that in the wet season males increased the singing intensity more (3.4 times; daily singing activity index in dry season was 14.3 ± 15.89 while in wet season 48.1 ± 35.05 songs per recording location per day) than females did (2.3 times; daily singing activity index in dry season was 2.6 ± 4.03 while in wet season 6.1 ± 6.78 songs per recording location per day) (Fig. 2). We did not find a significant effect of precipitation or interaction between sex and precipitation on the singing intensity (Table 1).

**Table 1:** Results of GLMM examining the effect of sex, season and precipitation on the daily singing activity index (the total number of songs detected in 56–58 1-min sound samples analysed per day).

Coefficients	Estimate	SE	Z	*p*
Intercept	2.886	0.493	5.85	** < 0.001**
Sex [male]	− 1.442	0.235	− 6.13	** < 0.001**
Precipitation	− 0.016	0.016	− 1.01	0.314
Season [Dry]	1.524	0.312	4.89	** < 0.001**
Sex*Precipitation	0.012	0.006	1.90	0.057
Sex*Season	− 0.365	0.139	− 2.63	**0.009**

Data were fitted by zero-inflated negative binomial distribution and log-link function. Significant results are in bold.

**Figure 3:**
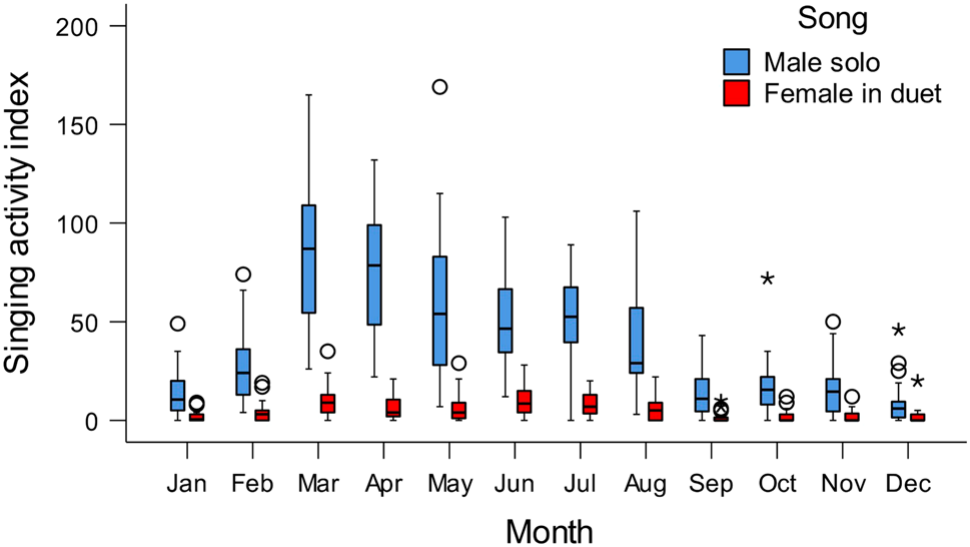
Changes in daily singing activity index of males and females across the year. Females never sing solo, therefore female songs are identical to duet. Medians, 1st and 3rd quartiles, maximum and minimum values, outliers (circles; observations that fall outside the expected range of data) and extremes (asterisks; observations that have unusually high or low values within the dataset) are given.

**Figure 4:**
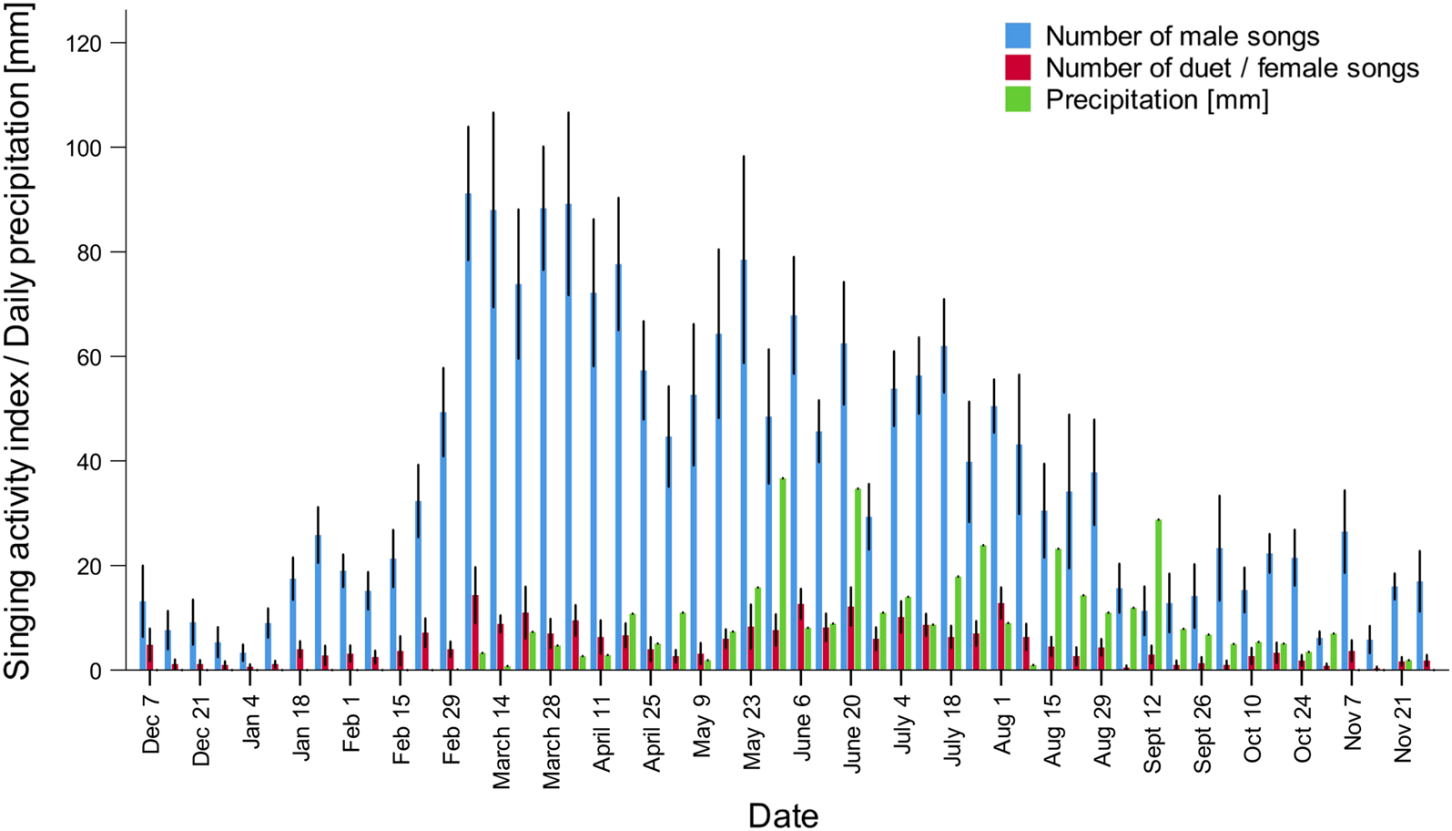
Yearly changes in daily singing activity index of males and females/duets (mean +/− SE) in relation to daily precipitation. Exact dates of recordings are given.

The peak of vocal activity of males was observed in March and April (daily singing activity index: 85.3 ± 33.99and 74.1 ± 33.35 songs per recording location per day, respectively), at the beginning of wet season (Fig. 3; Supplementary materials S2). The rapid increase of singing intensity was observed after the first rain and the high singing intensity was maintained until mid-April, when rainfall was still low (Fig. 4). After that, the daily singing activity index decreased, with the lowest values observed in December (daily singing activity index: 8.8 ± 10.99 songs per recording location per day). Females sang most intensively from March to August, with the highest value of vocal activity index observed in March (10.3 ± 9.48 songs per recording location per day; Fig. 3; Supplementary materials S2). The lowest singing activity of females was observed in October (daily singing activity index: 1.5 ± 2.72 songs per recording location per day) (Fig. 3).

### Daily vocal activity

**Figure 5:**
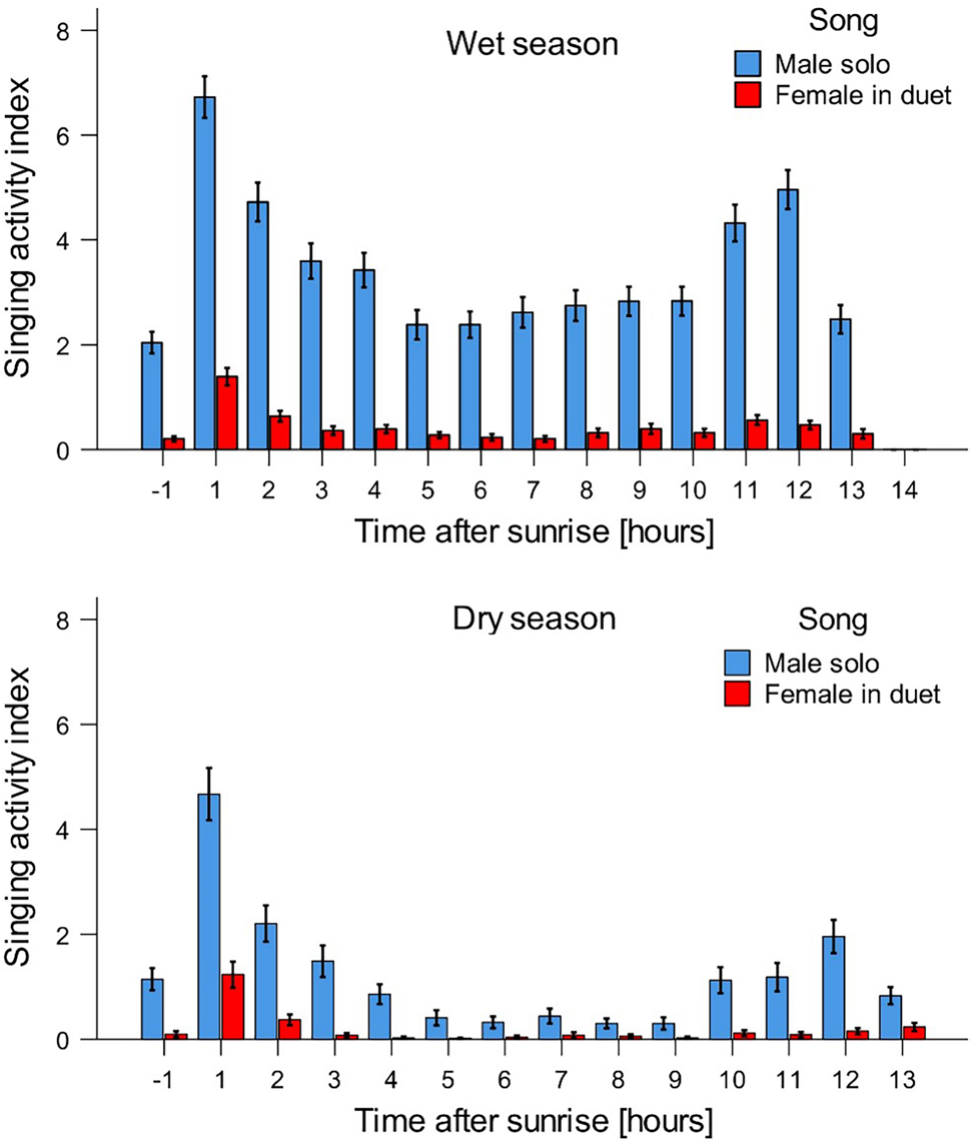
Daily changes in hourly singing activity index (the total number of songs detected in four 1 min sound samples analysed per hour per recording location) of males and females in wet (Mar–Oct) and dry (Nov–Feb) season. Mean values and standard errors for means are given.

Analysis of daily vocal activity pattern showed that Bangwa forest warblers sang more in wet than dry season, males sang more than females and singing intensity varied across a day. The daily activity pattern of singing was sex specific and seasonally variable (Table 2). Independently of a season males and females showed one, statistically distinguishable peak of vocal activity occurred in the first hour after sunrise—in this time they sang significantly more than during any other hour, independently on a season (Fig. 5; Supplementary materials S2). Males showed second, distinguishable peak of evening vocal activity observed in 11 (dry season), and 10 and 11 (wet season) hours after sunrise. In contrast, females did not show second peak of evening vocal activity. However, singing intensity during the morning peak (first hour after sunrise) was similar in wet and dry season (Mann–Whitney test; Z = − 1.277, *p* = 0.202), while during the rest of the day females sang significantly less in dry than in wet season (Fig. 5).

**Table 2:** Results of GLMM examining the effect of sex, time in day and season on daily vocal activity pattern.

Coefficients	Estimate	SE	Z	*p*
Intercept	2.171	0.415	5.23	** < 0.001**
Sex [male]	− 1.908	0.204	− 9.36	** < 0.001**
Hour	0.033	0.014	2.45	**0.014**
Season [Dry]	0.498	0.233	2.14	**0.033**
Sex*Hour	0.061	0.010	− 5.96	** < 0.001**
Sex*Season	0.247	0.112	2.21	**0.027**

As a dependent variable we used hourly singing activity index (the total number of songs detected in four 1-min sound samples analysed per hour per recording location). Data were fitted by zero-inflated negative binomial distribution and log-link function. Significant results are in bold.

## Discussion

Daily and seasonal singing activity patterns of male Bangwa forest warbler are similar to many temperate and tropical songbirds, with males singing most intensively around sunrise and showing seasonal peaks of vocal activity related to breeding activity^16^. Therefore, the Bangwa forest warbler can be classified as a truly sedentary species because males use songs for territory occupancy signalling all year round, and, in contrast to what was previously thought^32^, also classified as a typical wet season breeder, because the peak of singing activity of males is observed in wet season. However, in our study we did not mark birds individually. Therefore, although we observed that the territories are occupied year-round, we cannot be sure that the same birds are present within a territory throughout the year. The lack of knowledge about site fidelity and durability of pair bonds in Bangwa forest warbler limits the interpretation of seasonal peak of males’ vocal activity. The question of whether its main function is female attraction, resource defence, extra-pair copulations or something else still remains.

Results of this part of the study also demonstrate the value of autonomous sound recorders in revealing the aspects of biology and ecology of bird species which are difficult to observe otherwise, particularly those living in harsh and remote habitats. Probably our knowledge of the timing of breeding in many tropical species is too general. The results of this study demonstrate how the use of an acoustic approach allow us to track bird breeding phenology. Moreover, the information on seasonal variation in male singing rates can be used in species monitoring, indicating the optimal time for surveys, and explaining why in some periods of the year the probability of detection of Bangwa forest warbler and other sedentary tropical bird species is low and highly variable^30,33^.

In the Bangwa forest warbler females sing sex-specific song, however they do so at a significantly lower rate than males. Similar patterns of sex-specific songs and/or males singing more than females have been observed in many duetting species^26,34,35^, and may suggest different sex-role, various individual motivations and selective pressures which lead to evolution and maintenance of cooperative singing^36,37^. However, in Bangwa forest warbler females never sang solo and always joined a singing male to create duets, which means that only the females decided when to coordinate their songs with a male. Such female singing behaviour rules out several possible functions of duets and female songs in the study species. For example, when females never sing solo, they cannot use songs in their classical meaning to attract males, both in the context of pair bonding or extra-pair copulation. However, joining a male song can be a part of attraction process by signalling interest in pairing. The lack of solo songs also excludes intersex territory defence, in which females should use songs to defend resources against other females^38^. Duets observed year-round suggest that the function of cooperative singing could be a joint resource defence^20^. However, we do not yet know whether both sexes participate in territory defence and whether they do it jointly. Further studies using playback experiments are needed to support this hypothesis^39^.

The observed singing behaviour, where females sing only duets, suggests that the main functions of a female song in Bangwa forest warbler could be mate guarding, signalling commitment or defence of other resources^20^. By answering their partner's song, the females can advertise the mated status of their partner. In this way, the females may prevent their partners from being usurped, and maintain monogamy by minimising the chance for extra-pair copulation or for the male to take additional females. These hypotheses are also supported by seasonal pattern of females singing activity—females sing the most intensive from March to August, when males are also highly vocally active and show peak of seasonal vocal activity in the same month as males. Signalling commitment is especially important in sedentary species with a long-term partnership, where reproductive success depends on the effort of both partners. Therefore, in Bangwa forest warbler females responding to male solo songs all year round may be signalling the willingness to put effort in territory defence and other aspects of the partnership but also elicit ongoing investment from their partners. In some sedentary, socially monogamous and duetting bird species with long-term pair bonds the level of extrapair paternity can be high^40^, therefore mechanisms reducing extrapair paternity should be expected. However, further studies are needed to confirm and indicate which form of mate guarding, signalling commitment or defence of another resource is observed in Bangwa forest warbler, as has been done in Steere's liocichla *Liocichla steerii*^34^. Nevertheless, the fact that female songs are produced consistently, at a low rate throughout the year may suggest that female song has a similar function throughout the year, that loss of some resource (including mate) is equally likely and equally detrimental throughout the year or that the song serves multiple functions not exclusively tied to breeding^25^.

In many duetting species, the highest rate of duets has been observed during the first hours of the day^11,41–43^. In our study, we found a similar pattern since males and females sang most intensively in the first hour after sunrise. However, in Bangwa forest warbler males sang less at dawn in the dry than in wet season, while female singing rate during the morning chorus was seasonally stable. Moreover, unlike the males, females sang at more consistent rates during the whole day, and did not show a distinguishable second daily peak of vocal activity at dusk. There are several explanations for dawn chorus. Still, the best-supported hypotheses propose that singing in the morning has a relatively low energetic cost, is optimal for manipulating female mating, settle territory boundaries and may promote a handicap mechanism that prevents dishonest signalling^6^. The intensive all year round singing by females in the morning also supports the hypothesis that the main function of the female song could be mate guarding. However, we observed inter-territory differences in the proportion of duets, suggesting that females may be variously motivated to guard their mates. On the other hand, relatively constant rate of female song throughout the whole day suggests that other functions related to defence of resources other than a mate are also highly probable.

Female Bangwa forest warblers followed the male general seasonal singing activity pattern, however, we observed constantly high vocal activity from March to August, which was not observed in males. Assuming that the main function of female song in our study species is mate guarding and/or signalling commitment, we may expect that the first peak of vocal activity of females observed exactly in the same month like in males may be related to breeding activity, when both males and females sing intensively during a pre-breeding and breeding period^43^. The high vocal activity of females observed in the next months can be a response to increasing chance of male usurpation by females which lost their broods or young females which disperse and look for mates.

## Methods

### Study area

The study was conducted in Bamenda Highlands, near the Big Babanki village (North-West Region of Cameroon, coordinates: N6.09020°, E10.29461°). Bamenda Highlands are one of the biodiversity hotspots of endemism of regional and global importance located along border between Nigeria and Cameroon^28^. The climate is characterized by high rainfall (ca 2200 mm per year), and two seasons – rainy from March to October and dry from November to February^44^. Most of bird species in Bamenda Highlands breed in the dry season^32^. However, in some highland species a reversal of breeding season at lower altitudes is observed (i.e., the same species breeds in the dry season in highlands and in wet season in lowlands). Such breeding behaviour suggests high plasticity to weather conditions and clear seasonality of breeding^44^.

Our study area covered ca 2 km^2^ of unprotected mosaic of highland habitats (from 2050 to 2200 m asl), including montane forest, *Gnidia glauca* woodlands, stream buffers, grazing land, bush undergrowth and left-over plots dominated by bushes and *Pteridium aquilinum.* Human activity within the study area was minimal and limited to extensive grazing of cattle and horses.

### Soundscape recording

We collected soundscape recordings at six recording locations, from 7th December 2015 to 28th November 2016, using six Song Meter SM3 (Wildlife Acoustics) autonomous sound recorders equipped with SMM-A1 built-in omnidirectional microphones (signal-to-noise ratio > 68 dB). Recorders worked from one hour before sunrise to one hour after sunset, every seventh day (in total 52 days of recording at each recording location; 14–15 h of recordings per day, depending on time of sunrise and sunset; one-hour wav files, 48 kHz/16-bit sampling rate, 24 dB gain, no high or low pass filters applied). The recorders were placed on trees or shrubs, 2–4 m above the ground, depending on the habitat conditions. Previous studies showed that detection distance of songbird songs by autonomous sound recorders range between 100 and 150 m^45^. In our study the distance between recorders ranged from 267 to 741 m, however the nearest recorders were placed on the opposite sides of a slope. Therefore, the chances of recording the same individual from different recording locations were marginal. In our study we did not mark the birds individually. Therefore, it is possible that at the same recording location we recorded different birds across the year, because of mortality or territory takeover. However, we did not focus on singing behaviour of individual pairs but we tried to find general population pattern, therefore it does not limit the interpretation of our results.

### Bioacoustics analyses

We analysed 1-min sound samples to obtain the singing activity index of Bangwa forest warbler. From each 1 h soundscape recording we analysed four 1-min sound samples (1st, 15th, 30th and 45th min of recording). Therefore, hourly or daily singing activity index indicates the total number of songs recorded in four 1 min sound samples in an hour in a recording location, or the total number of songs recorded in all 1-min sound samples during a day in a recording location, respectively. In total we analysed 2960 min per recording location (6.7% of total soundscape data). Each 1 min sound sample was analysed by manual spectrogram scanning and listening to recordings in Raven Pro 1.6 software (Hann window, frame size 512, 3 dB filter, bandwidth 61.9 Hz, overlap of frames 50%). Each detected song of Bangwa forest warbler was classified into one of the three categories: male solo, female solo or duet (Fig. 1). When we classified the song as a duet, we also determined who initiated the duet: a male by joining a female solo song or a female by joining a male solo song (Supplementary Audio 2).

### Statistics

To examine seasonal changes in vocal activity of males and females in relation to precipitation we conducted Generalized Linear Mixed Models (GLMM). In the model as a dependent variable we used daily singing activity index, which was a sum of songs detected in all 1 min sound samples during a day at recording location (we analysed from 56 to 58 1 min sound samples per day per recording location, depending on day length). As categorical fixed effects we specified sex (male or female), season (dry or wet), precipitation in day of recording and two-way interaction terms of sex*precipitation and sex*season. The ID of recorder (six recorders) and day of recording (52 days) were included as a crossed random effect. Data were fitted by zero-inflated negative binomial distribution and log-link function. Data on rainfall were obtained from the nearest meteorological station located in Bamenda city (coordinates: N 5.964259°, E 10.161893°; 1250 m asl.; 20 km to the south-west from our study area). We used rainfall data from the exact same days in which we recorded the soundscape (daily sum of precipitation, in mm).

Similar GLMM was created to examine daily pattern of vocal activity. As a dependent variable we used the hourly singing activity index which was the sum of songs recorded in four 1 min sound samples in each hour (beginning one hour before sunrise, end one hour after sunrise; the last hour differed in the number of analysed 1 min sound samples because of varying day length). We specified sex (male or female) and season (dry or wet) as categorical and time of day (hour from sunrise) as a continuous fixed effect, and two-way interaction terms of sex*season and sex*time of day. We also included recorder ID and day of recording as a crossed random factor. Data were fitted by zero-inflated negative binomial distribution and log-link function.

To identify seasonal and daily peaks of vocal activity, we conducted additional GLMMs, separately for males and females. These models included the daily or hourly vocal activity index as a dependent variable, month or hour as a fixed categorical effect, and point ID as a random effect. In each model we used month or hour with the highest number of songs as a reference category. The data were fitted using a binomial distribution and a log-link function. The statistical analyses were conducted in R v. 4.2.1 using glmmADMB package v. 0.8.3.3. All reported p-values are two-tailed, all data are shown as mean +/– SE unless stated differently.

### Ethics approval

We got permission from the Kedjom-Keku community to conduct the study, including access to the study site and permission to camp in the mountains. The study was strictly observational, therefore additional permissions for conducting the study were not required.

## Supplementary Information

Supplementary Audio 1.

Supplementary Table S1.

Supplementary Table S2.

Supplementary Audio 2.

## Data Availability

All data generated or analysed during this study are included in this published article and its supplementary information. 1-min sound samples recordings used in the study are available from the corresponding author on reasonable request (m.budka@amu.edu.pl).
