# Supplementary material for: Females occasionally create duets with males but they never sing solo-year-round singing behaviour in an Afrotropical songbird
Source: Sci Rep. 2023 Jul 14;13:11405. doi: 10.1038/s41598-023-38552-5 (PMC10349113; doi:10.1038/s41598-023-38552-5)

Supplementary materials S2

S2 Table 1. Results of GLMMs identifying seasonal peaks of vocal activity of males and females of Bangwa forest warbler. In the model daily vocal activity index (number of songs produced per day per recording location) was defined as dependent variable, month as a fixed categorical effect, and point ID as a random effect. Month with the highest vocal activity index (March) was applied as a reference category. The data were fitted by using a binomial distribution and a log-link function. Significant differences are in bold.

Model term	Coefficient	SE	t	p
		Males vocal activity index		
Intercept	4.431	0.1833	24.178	**<0.001**
Month [Jan]	-1.771	0.2080	-8.513	**<0.001**
Month [Feb]	-1.055	0.1936	-5.450	**<0.001**
Month [Apr]	-0.172	0.2022	-0.851	0.396
Month [May]	-0.430	0.1922	-2.236	**0.026**
Month [Jun]	-0.531	0.2029	-2.619	**0.009**
Month [Jul]	-0.511	0.2028	-2.521	**0.012**
Month [Aug]	-0.844	0.1930	-4.372	**<0.001**
Month [Sep]	-1.910	0.2091	-9.135	**<0.001**
Month [Oct]	-1.593	0.1957	-8.139	**<0.001**
Month [Nov]	-1.690	0.2075	-8.146	**<0.001**
Month [Dec]	-2.317	0.2131	-10.873	**<0.001**
		Females vocal activity index		
Intercept	2.258	0.3240	6.968	**<0.001**
Month [Jan]	-1.430	0.3757	-3.806	**<0.001**
Month [Feb]	-0.884	0.3454	-2.559	**0.011**
Month [Apr]	-0.502	0.3603	-1.394	0.164
Month [May]	-0.543	0.3417	-1.588	0.113
Month [Jun]	-0.015	0.3561	-0.043	0.966
Month [Jul]	-0.320	0.3585	-0.893	0.373
Month [Aug]	-0.600	0.3422	-1.752	0.081
Month [Sep]	-2.072	0.3967	-5.223	**<0.001**
Month [Oct]	-1.694	0.3606	-4.699	**<0.001**
Month [Nov]	-1.772	0.3855	-4.598	**<0.001**
Month [Dec]	-1.753	0.3848	-4.555	**<0.001**

S2 Fig. 1. Changes in daily singing activity index of (a) males and (b) females across the year. Medians, 1st and 3rd quartiles, maximum and minimum values, outliers (circles; observations that fall outside the expected range of data) and extremes (asterisks; observations that have unusually high or low values within the dataset) are given.

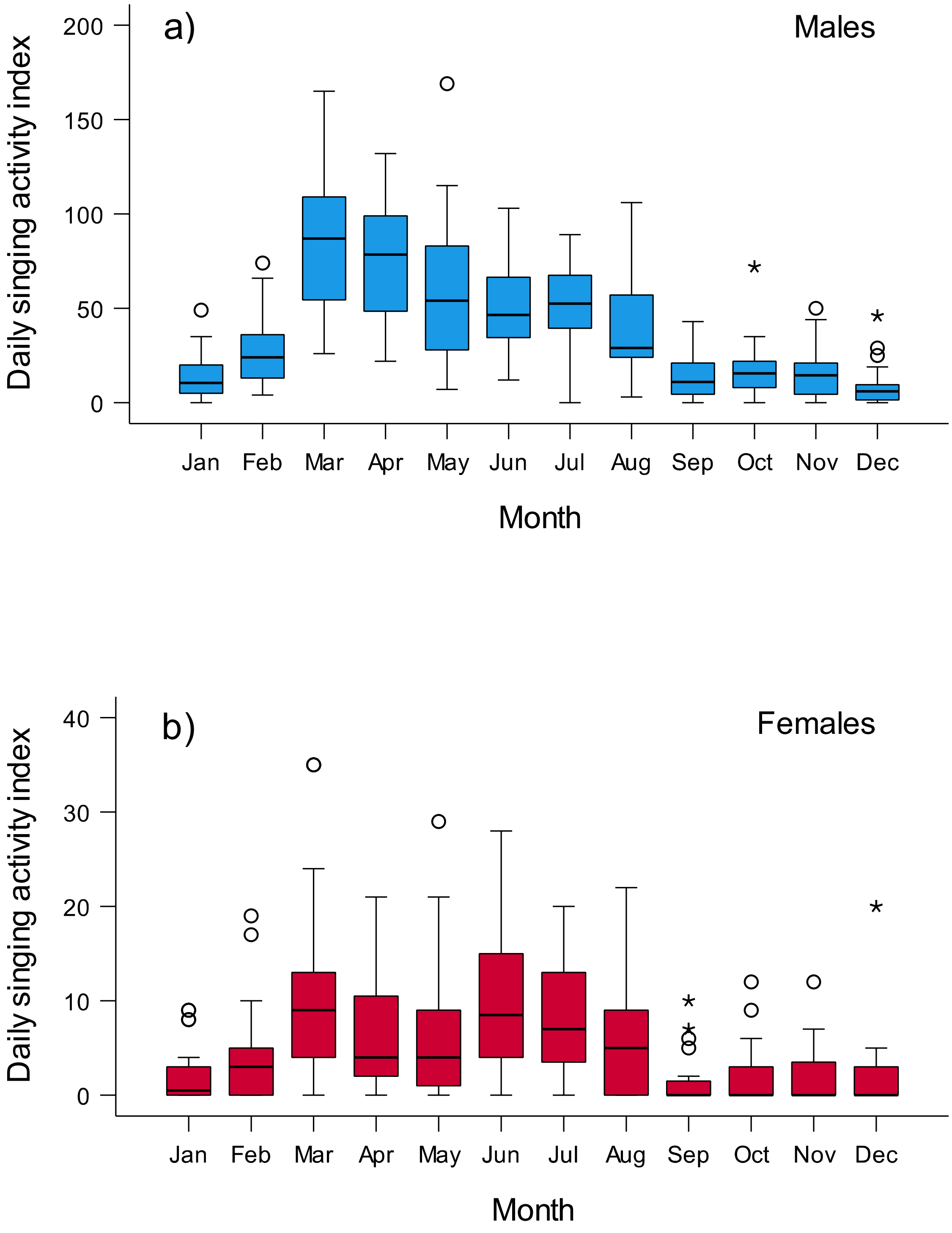

S2 Table 2. Results of GLMMs identifying daily peaks of vocal activity of males and females of Bangwa forest warbler. In the model vocal activity index (number of songs produced per hour per recording location) was defined as dependent variable, hour as a fixed categorical effect, and point ID as a random effect. Hour with the highest vocal activity index (0) was applied as a reference category. The data were fitted by using a binomial distribution and a log-link function. Significant differences are in bold.

Model term	Coefficient	SE	t	p
		Males vocal activity index		
Intercept	1.787	0.1709	10.457	**<0.001**
Hour [-1]	-1.139	0.1604	-7.100	**<0.001**
Hour [1]	-0.458	0.1577	-2.906	**<0.01**
Hour [2]	-0.753	0.1586	-4.745	**<0.001**
Hour [3]	-0.911	0.1593	-5.720	**<0.001**
Hour [4]	-1.300	0.1613	-8.057	**<0.001**
Hour [5]	-1.297	0.1613	-8.041	**<0.001**
Hour [6]	-1.216	0.1608	-7.562	**<0.001**
Hour [7]	-1.201	0.1607	-7.474	**<0.001**
Hour [8]	-1.160	0.1605	-7.227	**<0.001**
Hour [9]	-1.029	0.1598	-6.438	**<0.001**
Hour [10]	-0.639	0.1583	-4.040	**<0.001**
Hour [11]	-0.455	0.1577	-2.884	**<0.01**
Hour [12]	-1.143	0.1604	-7.125	**<0.001**
		Females vocal activity index		
Intercept	0.241	0.2206	1.091	0.275
Hour [-1]	-2.079	0.1666	-12.480	**<0.001**
Hour [1]	-0.918	0.1227	-7.482	**<0.001**
Hour [2]	-1.644	0.1458	-11.277	**<0.001**
Hour [3]	-1.670	0.1468	-11.371	**<0.001**
Hour [4]	-2.003	0.1625	-12.327	**<0.001**
Hour [5]	-2.111	0.1683	-12.538	**<0.001**
Hour [6]	-2.128	0.1693	-12.567	**<0.001**
Hour [7]	-1.801	0.1526	-11.805	**<0.001**
Hour [8]	-1.649	0.1460	-11.298	**<0.001**
Hour [9]	-1.714	0.1487	-11.524	**<0.001**
Hour [10]	-1.250	0.1317	-9.490	**<0.001**
Hour [11]	-1.323	0.1340	-9.873	**<0.001**
Hour [12]	-1.601	0.1440	-11.115	**<0.001**

S2 Fig. 2. Changes in hourly singing activity index of (a) males and (b) females. Mean values (+/- SE) are given.

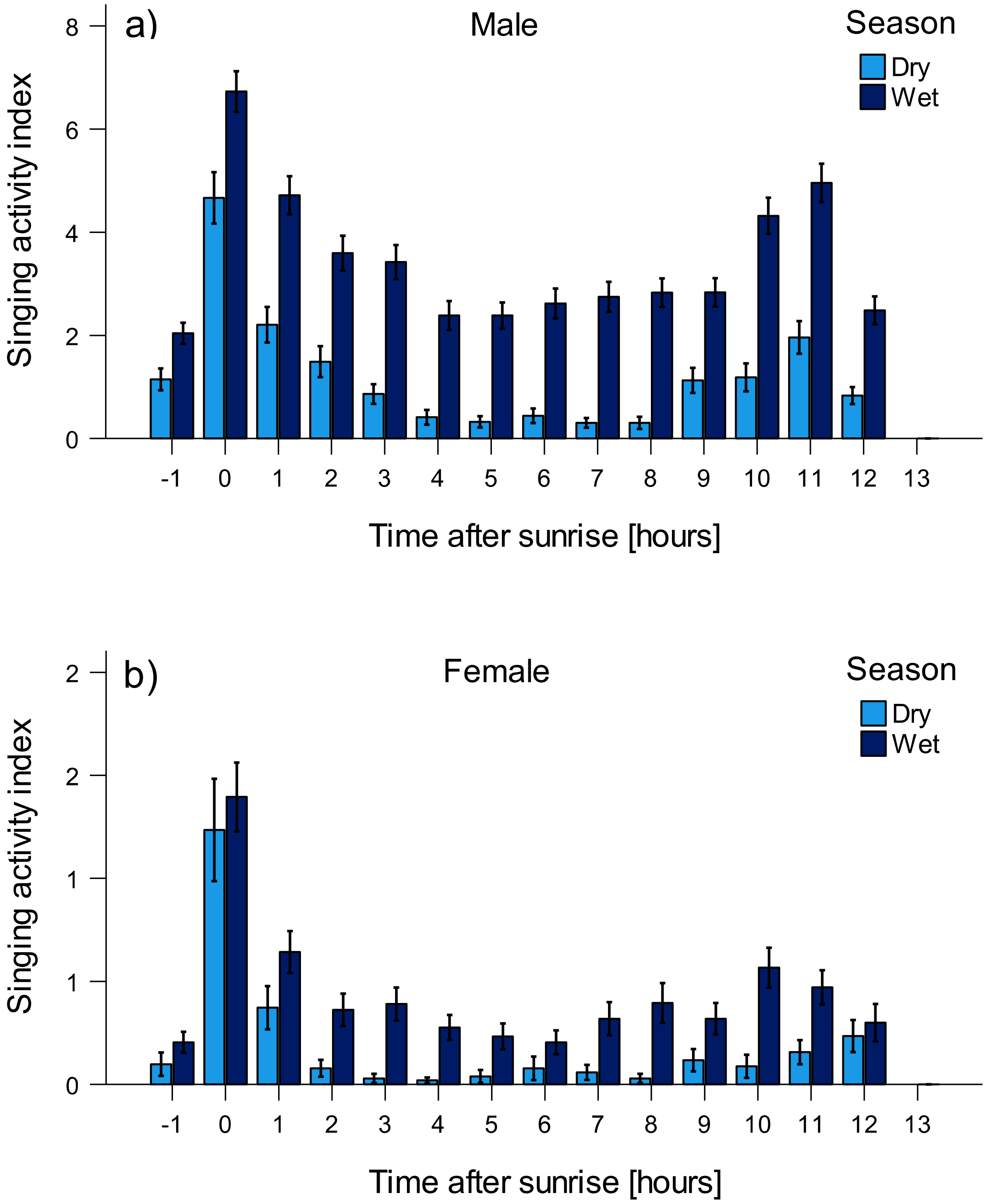

Supplement: Supplementary file 3 — Supplementary Table S2. [file 41598_2023_38552_MOESM3_ESM.pdf]
